# Vasorin-deficient mice display disturbed vitamin D and mineral homeostasis in combination with a low bone mass phenotype

**DOI:** 10.1016/j.bonr.2024.101792

**Published:** 2024-07-18

**Authors:** Marco Eijken, A. Michaela Krautzberger, Manuela Scholze-Wittler, Bianca Boers-Sijmons, Marijke Koedam, Barbara Kosiol, Heinrich Schrewe, Johannes P. van Leeuwen, Bram C. van der Eerden

**Affiliations:** a Internal Medicine, Erasmus MC, Rotterdam, the Netherlands; b Department of Developmental Genetics, Max Planck Institute for Molecular Genetics, Berlin, Germany; c Department of Clinical Medicine, Aarhus University, Denmark

**Keywords:** Vasorin, Vitamin D, Vitamin D binding protein, Bone, Osteoblast

## Abstract

Vasorin (Vasn) is a pleiotropic molecule involved in various physiological and pathological conditions, including cancer. Vasn has also been detected in bone cells of developing skeletal tissues but no function for Vasn in bone metabolism has been implicated yet. Therefore, this study aimed to investigate if Vasn plays a significant role in bone biology. First, we investigated tissue distribution of *Vasn* expression, using lacZ knock-in reporter mice. We detected clear Vasn expression in skeletal elements of postnatal mice. In particular, osteocytes and bone forming osteoblasts showed high expression of Vasn, while the bone marrow was devoid of signal. Vasn knockout mice (*Vasn*^*−/−*^) displayed postnatal growth retardation and died after four weeks. MicroCT analysis of femurs from 22- to 25-day-old *Vasn*^*−/−*^*mice* demonstrated reduced trabecular and cortical bone volume corresponding to a low bone mass phenotype. *Ex vivo* bone marrow cultures demonstrated that osteoclast differentiation and activity were not affected by *Vasn* deficiency. However, osteogenesis of *Vasn*^−/−^ bone marrow cultures was disturbed, resulting in lower numbers of alkaline phosphate positive colonies, impaired mineralization and lower expression of osteoblast marker genes. In addition to the bone phenotype, these mice developed a vitamin D_3_-related phenotype with a strongly reduced circulating 25-hydroxyvitamin D_3_ and 1,25-dihydroxyvitamin D3 and urinary loss of vitamin D binding protein. In conclusion, Vasn-deficient mice suffer from severe disturbances in bone metabolism and mineral homeostasis.

## Highlights

• Mice lacking vasorin have a low bone mass phenotype.
• Mice lacking vasorin suffer from disturbed vitamin D and mineral homeostasis.
• Mice lacking vasorin display growth retardation and die after 4 weeks of age.

## 1 Introduction

Vasorin (Vasn) is a typical type I cell surface transmembrane glycoprotein of about 110 kDa (Bonnet et al., 2018). Vasn was first localized in adult human tissues in 2004, implicating a role in the regulation of TGFß signaling (Ikeda et al., 2004). Since then, several publications established an association between Vasn and the development of pathological conditions, including different types of cancer (Liang et al., 2023; Man et al., 2018; Yeo et al., 2019).

It was recently reported that CrispR-Cas9-mediated Vasn-deficient mice suffered from malnutrition, a shortened lifespan, and affected physiological functions related to liver injury (Yang et al., 2022). Moreover, Vasn deficiency has been associated with lung injury through inflammatory mechanisms as well as with cardiac hypertrophy and arterial function (Guo et al., 2022; Sun et al., 2022; Louvet et al., 2022). Analyses using a *lacZ* knock-in reporter line demonstrated widespread Vasn expression throughout embryonic development with clear expression in the developing skeletal tissues (Krautzberger et al., 2012).

During adulthood bone tissue is continuously rebuilt by bone forming osteoblasts and bone resorbing osteoclasts. The bone-embedded osteocytes can send signals to osteoblasts and osteoclasts through cellular canaliculi in situations of mechanical loading of bone or musculoskeletal disuse (Komori, 2013). This intricate cellular network requires complex interaction between the different bone cells but also with the extracellular bone matrix components within the bone microenvironment.

The prominent reporter expression observed in fetal developing bones of Vasn^*lacZ/+*^ mice prompted a more detailed analysis of Vasn function in bone. In this study, we examined *Vasn* expression in neonatal bone tissue and studied the impact of *Vasn* deficiency on bone metabolism as well as calcium and phosphate homeostasis using global *Vasn* knockout mice.

## 2 Material and methods

### 2.1 Animal housing

Mice were kept at the Max Planck Institute for Molecular Genetics in Berlin, and all animal experiments conformed to the German Authority regulations. The Max Planck Institute for Molecular Genetics holds licenses that have been approved by the Landesamt für Gesundheit und Soziales (LAGeSo) in Berlin (Licenses: G0368/08 and ZH 120). Animals were housed in a specific pathogen free (SPF) facility with a 12 h day-night cycle in a controlled room with temperatures of 22 ± 1 °C and humidity of 50 ± 5 %. Mice were fed with irradiated standard and breeding rodent diet *ad libitum*.

### 2.2 Generation of Vasn-deficient mice

Generation of Vasn deficient mice is depicted in Supplementary Fig. 1. To elucidate the function of Vasn *in vivo*, we targeted the *Vasn* locus in G4 embryonic stem (ES) cells (Guo et al., 2022) in an approach that allows *loxP*/Cre-mediated excision of the coding region-containing exon 2. The plasmid used for targeting the mouse Vasn locus harbored a 10.5 kb-long construct inserted into the pBluescript II SK vector (Stratagene). The insert contained a loxP-flanked (PGK-driven) neomycin selection cassette in the intronic sequence of Vasn 68 bp upstream of the ATG start codon on exon 2 and a third loxP site 96 bp downstream of the TAG stop codon in the 3′ untranslated region (UTR). This modified version of the *Vasn* coding exon 2 was flanked by a 3 kb- and a 3.6 kb-long 5′ and 3′ arm of homology, respectively. The complete targeting vector was linearized with *Nru*I prior to electroporation into G4 ES cells (male F1 hybrid cells of maternal 129S6/SvEvTac and paternal C57Bl/6Ncr background). Neomycin-resistant ES cell colonies were screened for integration of the construct at the correct locus by Southern blot analysis of *Hin*dIII-digested genomic DNA using 5′ and 3′ external probes. The introduction of an additional HindIII site downstream of the third loxP site allowed for discrimination between the wild type and targeted Vasn^neo-flox^ allele. Successfully targeted Vasn^neo-flox/+^ ES cells were transiently transfected with a plasmid expressing Cre recombinase under control of the CAG promoter (pTurbo-Cre; kindly provided by Dr. Timothy Ley, Washington University, St. Louis, MO, USA) to excise loxP-flanked sequences. Derived ES cell colonies were again screened by Southern blot analysis as described above to identify clones carrying a Vasn^flox^ or Vasn^null^ allele. Chimeric male mice were generated by aggregating Vasn^null/+^ and Vasn^flox/+^ ES cells with wild type CD-1 morulae and backcrossed to C57Bl/6 females to obtain Vasn^null/+^ and Vasn^flox/+^ offspring. Heterozygous Vasn^null/+^ and Vasn^flox/+^ mice were intercrossed to obtain homozygous knockout animals, as well as homozygous conditional knockout mice that behaved as wild type (data not shown). Heterozygous *Vasn*^*+/−*^ animals, carrying one wild type and one null allele, were intercrossed, and wild type, heterozygous, and homozygous offspring were identified by PCR. Genotyping was performed by PCR on DNA extracted from tail biopsies using a coding sequence (5’-GGCAACTTCTACAGCTCAGG-3′) or intron (5’-GACCTGACTCACACGTTCTGGG-3′) forward primer for the wild type or null allele, respectively, and a common reverse primer (5’-AGATGAGACCCAGCCCAGAG-3′). PCR products were analyzed on a 2 % agarose gel to resolve the 348 bp and 146 bp amplicons from the wild-type and mutant alleles, respectively. Total RNA was isolated from freshly dissected kidneys using the RNeasy® Midi Kit (Qiagen) according to the manufacturer's instructions. Northern blot analysis was performed with 10 μg total RNA from kidney of *Vasn*^*+/−*^ and *Vasn*^*−/−*^ mice using a probe corresponding to the 3’ UTR of *Vasn*.

### 2.3 X-gal staining of bone sections

Embryos were embedded as described before (Krautzberger et al., 2012). Tibiae were cleared from soft tissue and decalcified in OSTEOSOFT® solution (Merck) overnight rolling at RT. The next day, the bones were incubated in Tissue-Tek OCT compound (Ted Pella) for 1 h at 4 °C before embedding them in molds filled with freezing compound on a metal block cooled with dry ice and ethanol. Eight μm-thick sections of embryos and tibiae were cut and stained for β-galactosidase activity. Briefly, the sections were postfixed on ice in 1 % PFA/PBS for 10 min before quickly rinsing and then washing them on ice in cold 2 mM MgCl_2_/PBS for 10 min on a shaker. After two washes with cold rinse buffer for 10 min, the slides were incubated in staining buffer o/n in the dark at 37 °C. The next day, the sections were washed three times in 2 mM MgCl_2_/PBS for 5 min at RT, rinsed in ddH_2_O for 5 min, counterstained, dehydrated, and mounted.

### 2.4 Microcomputed tomography (μCT) analysis

Femurs were scanned *ex vivo* at a resolution of 9 μm, using a SkyScan 1076 system (Bruker microsystems, Kontich, Belgium) in a blinded fashion. According to guidelines recently published (Bouxsein et al., 2010), the following settings were used: X-Ray power and tube current were 40 kV and 250 μA, respectively. Beam hardening was reduced using a 1 mm aluminium filter, exposure time was 5.9 s and an average of three pictures was taken at each angle (0.9°) to generate final images. Segmentation of the reconstructed images was done on basis of global thresholding. Using different software packages from SkyScan (NRecon, CtAn and Dataviewer), bone microarchitectural parameters were assessed in trabecular and cortical bone. The trabecular bone parameters trabecular tissue volume, bone volume, trabecular bone volume fraction (BV/TV), trabecular thickness (Tb.Th), trabecular number (Tb.N), trabecular spacing (Tb.Sp), trabecular patterning factor (Tb.Pf) and structure model index (SMI) were determined in the distal metaphysis of the femur (scan area of 3.15 mm from distal growth plate towards femoral centre). In the mid-diaphysis (scan area of 0.9 mm in the femoral centre), cortical area (Ct.Ar), cortical thickness (Ct.Th), moment of inertia (MOI; proxy for bone strength) and periosteal perimeter (Ps.Pm) were analyzed.

### 2.5 Bone marrow cultures

Tibial and femoral bone marrow cells were collected using a centrifugation method. From each mouse, 1 femur and 1 tibia were cut at the diaphysis close to the knee joint. Bones were each placed into a 1.5 ml eppendorf tube containing a handmade plastic adaptor pre-filled with 250 μl culture medium. Bones were centrifuged at 1000 *g* and spun out bone marrow cell pellets were resuspended in 10 ml culture medium. Cells were centrifuged at 300 *g* for 5 min and resuspended in 1 ml culture medium followed by addition of 9 ml erylysis buffer (155 mM NH_4_Cl, 10 mM KHCO_3_, 0.1 mM EDTA) and incubated for 5 min on ice. Next, cells were washed twice with 10 ml of culture medium by centrifugation followed by counting the cells using a Casy cell counter (Schärfe System). Osteoblast cultures were started by seeding 5 × 10^5^ cells per 12-well plate well in 700 μl phenol-red free α-minimal essential medium (Invitrogen), supplemented with 100 units/ml penicillin, 100 μg/ml streptomycin (Invitrogen), 250 ng/ml amphotericin B (Sigma), 20 mM Hepes, 1.8 mM CaCl_2_, and 15 % (vol/vol) heat-inactivated FCS (Invitrogen), pH 7.5. Two days after seeding cultures were refreshed with medium supplemented with 50 μM vitamin C (Sigma) and 10 mM β-glycerophosphate (Sigma) twice a week. At days 4, 6 and 9 and days 11, 14 and 17 of culture cells were fixed in 70 % ethanol and stained for alkaline phosphatase (ALP) and alizarin red, respectively. For ALP staining, cells were incubated in Tris-HCl (pH 9.5) containing 50 mM MgCl_2_, 0.6 mg/ml bromo-chloro-indolyl phosphate (Sigma), and 150 μg/ml nitro blue tetrazolium (Sigma) for 20 min and washed with PBS. Alizarin red staining was performed, incubating the cells for 10 min in a saturated alizarin red solution in distilled water (pH 4.2), after which the cells were washed with distilled water. The number of ALP- and alizarin red-positive colonies was counted.

Osteoclast cultures were started by seeding 75 × 10^3^ cells per 96-well plate well. Cells were cultured for 6 days in the presence of 30 ng/ml recombinant M-CSF (R&D systems) and 20 ng/ml recombinant murine RANKL-TEC (R&D systems), the media were refreshed at day 3. At the end of the murine cultures, cells were washed with PBS, fixed in PBS-buffered paraformaldehyde (4 % *v*/v) or formalin (10 % v/v), respectively, and stored at 4 °C for tartrate-resistant acid phosphatase (TRACP) staining. To do so, we used a leucocyte kit (Sigma) according to manufacturer's protocol and counted TRAP-positive mono- bi- and multinucleated osteoclast numbers. In addition, murine osteoclasts cultured on bovine cortical bone slices for 6 days were lysed in water for Coomassie brilliant blue staining of resorption pits (Yang et al., 2022).

### 2.6 Quantitative PCR

Kidneys and livers of WT and *Vasn*^−/−^ mice were put in Trizol (Invitrogen) and homogenized using a TissueLyser (Qiagen). Osteoblast cultures (day 10) were washed with PBS followed by the addition of Trizol. Trizol-based RNA isolation was performed according to manufacturer's protocol. After RNA isolation, 1 μg of total RNA was converted to cDNA using a cDNA synthesis kit (MBI Fermentas) according to the protocol of the manufacturer, using 0.5 μg oligo(dT)_18_ and 0.2 μg random hexamer primers. qPCR was carried out using an ABI 7700 sequence detection system (Applied Biosystems, Foster City, CA). Reactions were performed in 25 μl volumes using a qPCR core kit or qPCR kit for SYBR green I (Eurogentec). Cycling conditions were 50 °C for 2 min, 95 °C for 10 min, followed by 40 cycles of 95 °C for 15 s and 60 °C for 1 min. Genes were quantified relatively against *Hprt1* expression. Primer and probes sequences and concentrations are listed in Supplementary Table 1.

### 2.7 Hormone levels

Serum FGF23 (C-Terminal) and PTH (Intact) were both measured using an ELISA kit (Immutopics) according to the manufacturer's protocol.

### 2.8 Vitamin D Binding Protein (DBP) measurements

Mouse urine DBP levels were measured by a direct ELISA using anti-hDBP sheep IgG antibody (R&D systems, AF4188). In total, 100 μl diluted urine (diluted to 0.05 mg/ml protein in carbonate buffer) was coated in a 96-wells maxisorp immunoplate plate (Nunc) over-night at 4 °C. Next day wells were washes twice using PBS and blocked with 200 μl PBS 1 % bovine serum albumin (BSA, Sigma) for 2 h at RT followed by washing the wells twice with PBS. Subsequently, wells were incubated with 100 μl 0.2 μg/ml anti-hDBP sheep IgG antibody (R&D systems, AF4188) in PBS 1 % BSA for 2 h at RT. Wells were washed 4 times with PBS 0.05 % tween-20 (Sigma) and incubated with 100 μl 0.2 μg/ml polyclonal rabbit a-sheep-immunoglobulins-HRP (Dako) in PBS 1 % BSA for 2 h at RT. Wells were washed 4 times with PBS 0.05 % tween-20 and incubated with 100 μl TMB liquid ELISA substrate (Sigma) for approximately 10 min until a blue color developed. Reaction was stopped by adding 50 μl 2 M H_2_SO_4_. Extinction was measured at 450 nm.

### 2.9 Calcium and phosphate levels

Serum and urinary calcium levels were colorimetrically determined. Ten μl of serum was added in a 96-wells plate (Greiner bio-one) and calcium was quantified by adding 100 μl calcium reagents (1 M ethanolamine buffer (pH 10.6) 0.35 mM o-cresolphtalein complexone, 19.8 mM 8-hydroxyquinoline and 0.6 mM hydrochloric acid). CaCl_2_ was used for a standard curve and samples were measured at 595 nm. Serum and urinary phosphate were measured using a Phosphorus liquid-UV® assay (Stanbio laboratory) according to manufacturer's protocol.

### 2.10 SDS-PAGE urinalysis

Total urinary proteins were detected by SDS-PAGE and subsequent staining with PageBlue™ Protein Staining Solution (Fermentas). Urine was collected by re- straining the mouse with one hand and gently pressing the thumb of the other hand against the lower abdomen. 2 μl urine were diluted in 13 μl buffer (10 mM Tris-HCl pH 8.0, 2.5 mM MgCl_2_, 5 mM EGTA pH 8.0). 5 μl 4× NuPAGE® LDS Sample Buffer (Invitrogen) were added, and the proteins were denatured for 10 min at 70 °C, then put on ice. The samples were loaded into the wells of a precast NuPAGE® 10 % Bis-Tris gel and electrophoretically separated for 1 h at 200 V in 1× NuPAGE® MOPS SDS Running Buffer. Residual SDS was removed by rinsing the gel in ddH_2_O. The proteins were visualized by incubating the gel in PageBlue™ Protein Staining Solution (Fermentas) for 1 h at RT on a shaker. Excess staining solution was removed by washing the gel o/n at RT in ddH_2_O on a shaker.

### 2.11 Statistics

Statistical calculations were done, using GraphPad Prism 5.0. In all experiments values were expressed as mean ± SEM unless stated otherwise. Differences between groups were tested for significance using the Student-*t*-test. Values were considered significantly different at *p* < 0.05. Statistical analyses revealed that genotype and sex showed no significant interaction for any parameter studied. Therefore, male and female mice were combined in all presented studies. No significant difference was observed between wild-type and heterozygous mice for any of the measured parameters. Wild-type and heterozygous mice were grouped and served as controls in the datasets presented in Fig. 4, Fig. 5, Fig. 6, Fig. 7, Fig. 8.

## 3 Results

### 3.1 Vasn is expressed in bone tissue

**Fig. 1:**
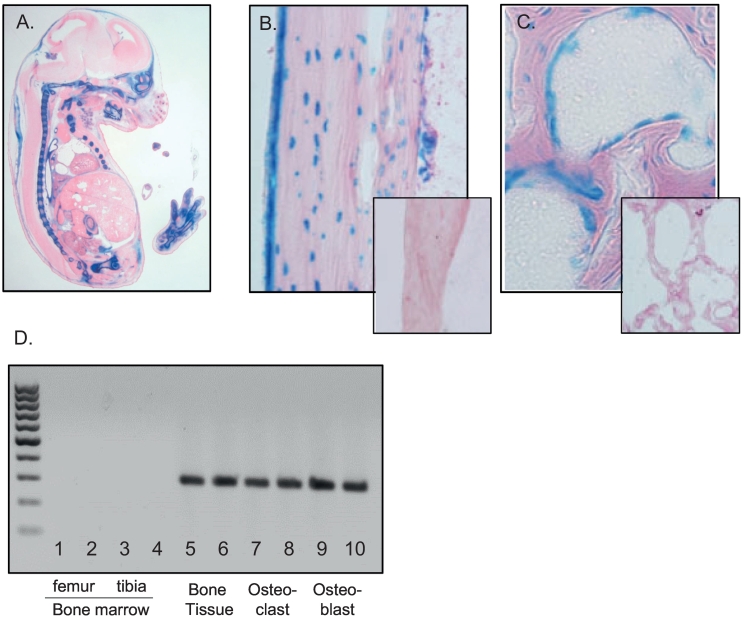
LacZ reporter activity in a *Vasn*^*lacZ*^ embryonic day E15.5 embryo, indicating widespread expression of Vasn, predominantly in the skeleton (A)**.** LacZ expression in periosteal osteoblasts and osteocytes (B) as well as trabecular osteoblasts (C) from 10-week-old *Vasn*^*lacZ*^ mice. Insets in B and C represent stainings on wild type mice. Vasn mRNA expression panel (D) for 2 samples of femoral bone marrow (slot 1 + 2), tibial bone marrow (slot 3 + 4), mouse bone (slot 5 + 6), mouse osteoclasts (slot 7 + 8), mouse osteoblasts (slot 9 + 10).

The generation of *Vasn*^lacZ^ knock-in reporter mice (Vasn^tm1.1Hsch^) has been reported previously (Man et al., 2018). Analyses demonstrated widespread reporter activity throughout mouse embryonic development, with expression being most prominent in the developing skeletal system, as shown for embryonic day 15.5 *Vasn*^lacZ^ embryos (Fig. 1A). To determine whether high-level expression of *Vasn* in skeletal structures persists postnatally, tibiae of 10-week-old *Vasn*^lacZ/+^ mice were sectioned and stained for β-galactosidase (β-gal) activity. As for embryonic stages, reporter expression was found to be strong in bone tissue at this age. LacZ staining was clearly detected in osteocytes located in cortical and trabecular bone structures (Figs. 1B and C). In addition, the periosteal and endosteal cell layers were intensely stained. The bone marrow was completely devoid of signal (Fig. 1B and C). RT-PCR on mature mice bones confirmed abundant *Vasn* expression in bone tissue. Osteoblast and osteoclast *in vitro* cultures derived from bone marrow of mature mice showed clear *Vasn* expression. Bone marrow was devoid of *Vasn* transcripts (Fig. 1D).

### 3.2 Generation of Vasn knock-out mice

Vasn knock-out mice (V*asn*^*−/−*^) were used to investigate the impact of Vasn on bone. While heterozygous mice were indistinguishable from wild type littermates, homozygous mice displayed postnatal growth retardation and died after four weeks after birth. In all studies, the mice were 22 to 25 days old.

### 3.3 Vasn-deficient mice (Vasn^−/−^) have a reduced bone mass phenotype

**Fig. 2:**
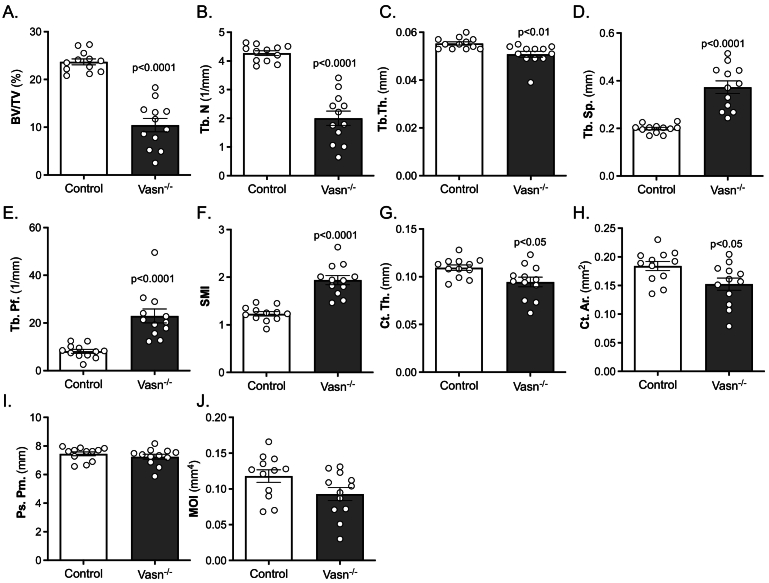
μCT analysis of wild type (Control) and *Vasn*^−/−^ mice (*n* = 12) femurs demonstrating trabecular bone volume fraction (BV/TV) (A), trabecular number n(Tb.N) (B), trabecular thickness (Tb.Th) (C), trabecular spacing (Tb.Sp) (D), trabecular pattern factor (Tb.Pf) (E), structure model index (SMI) (F), cortical thickness (Ct.Th) (G), cortical area (Ct.Ar) (H), periosteal perimeter (Ps.Pm) (I) and moment of inertia (MOI) (J).

Femurs of *Vasn*^−/−^ mice and wild type littermates (controls) were analyzed using μCT. This revealed significant changes in both bone volume and bone structure. *Vasn*^−/−^ mice displayed strongly decreased BV/TV (Fig. 2 A) predominantly due to a highly significant reduction in Tb.N (Fig. 2B) as Tb.Th was only slightly reduced (Fig. 2C). In line with the reduced BV/TV and Tb.N, Tb.Sp was increased (Fig. 2D), and connectivity was diminished as shown by a sharp increase in Tb.Pf (Fig. 2E). The SMI indicated a significant shift from a plate-like to a rod-like structure of the trabecular network (Fig. 2F). The changes observed in cortical bone structures were less pronounced. Both Ct.Th and Ct.Ar were slightly but significantly reduced (Fig. 2G and 2H), while Ps.Pm and MOI were not significantly different between the genotypes (Figs. 2I and 2 J).

**Fig. 3:**
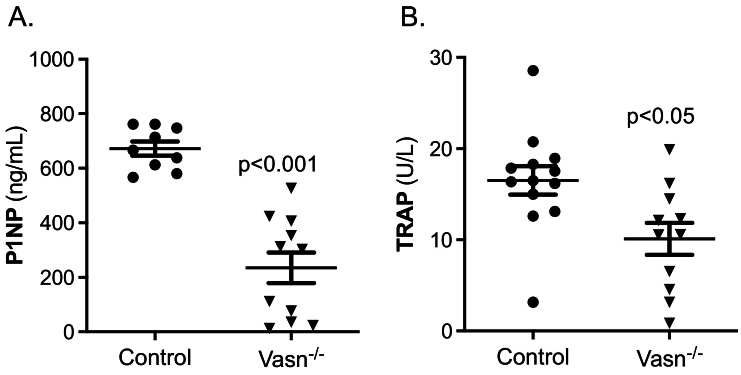
Bone formation (A) and resorption (B) markers total procollagen 1 N-terminal pro peptide (P1NP) and tartrate-resistant acid phosphatase (TRAP), respectively, determined in serum of wild type (control) and *Vasn*^−/−^ mice (P1NP: *n* = 9–11; TRAP: *n* = 11–13).

Effects on bone formation and resorption were assessed by measuring serum levels of amino-terminal propeptide of type 1 procollagen (P1NP) and tartrate-resistant acid phosphatase (TRAP), respectively. Serum P1NP was decreased by 65 % in *Vasn*^−/−^ mice compared to controls (Fig. 3A). Serum TRAP was reduced by approximately 30 % in *Vasn*^−/−^ mice (Fig. 3B).

### 3.4 Vasn-deficient osteoblast differentiation is impaired

**Fig. 4:**
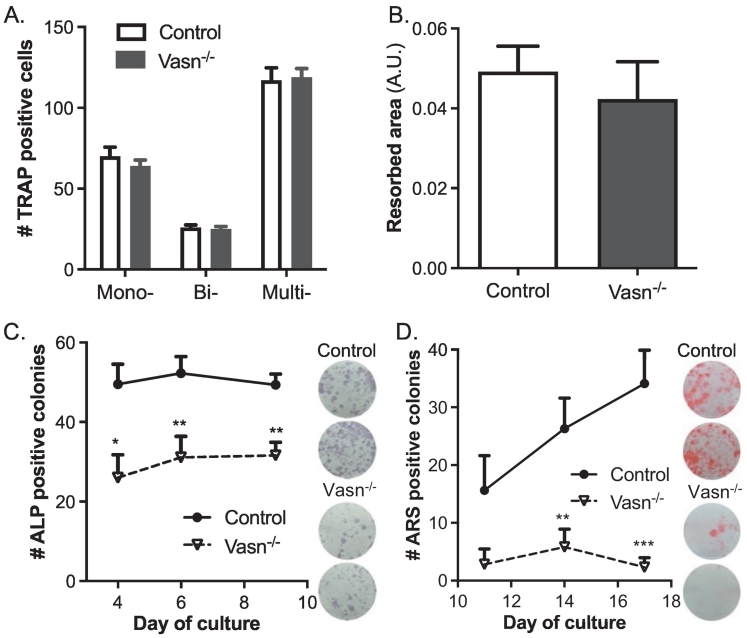
Osteoclastogenesis and osteoblastogenesis of bone marrow cultures derived from control and *Vasn*^−/−^ mice (*n* = 8). After 6 days of differentiation TRAP positive mono- bi- and multi-nucleated cells were counted (A) and bone resorption pits were stained and quantified (B). Osteoblast cultures were stained after 4, 6 and 9 days for quantification of alkaline phosphatase (ALP) positive colonies (C) and after 11, 14 and 17 days for mineralized colonies, using alizarin Red (D). *p < 0.05, **p < 0.01, ***p < 0.001.

Bone marrow cells from control and *Vasn*^−/−^ mice were cultured to study osteoblast and osteoclast function *ex vivo*. TRAP staining and bone resorption analyses demonstrated identical osteoclast potential in bone marrow cultures of control and *Vasn*^*−/−*^ mice (Fig. 4A and B).

**Fig. 5:**
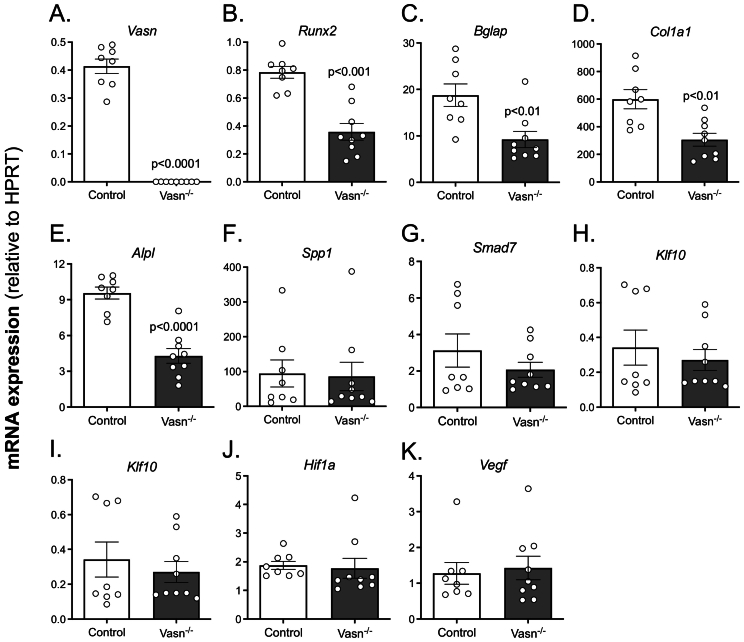
Gene expression in bone marrow-derived osteoblast cultures from control and *Vasn*^−/−^ mice. Osteoblastic mRNA expression of (A) *Vasn*, (B) *Runx2*, (C) *Bglap* (D) *Col1a1*, (E) *Alpl*, (F) *Spp1*, (G) *Smad7*, (H) *Klf10*, (I) *Sod1*, (J) *Hif1a* and (K) *Vegf*, all corrected by normalization for the housekeeping gene *Hprt1* (*n* = 8–9).

In contrast, significantly fewer alkaline phosphatase (ALP)-positive colonies were formed in osteogenic *Vasn*^−/−^ bone marrow cultures (Fig. 4C). In addition, there was significantly less formation of mineralized colonies (Fig. 4D). These findings were further supported by quantification of mRNA levels of osteoblast differentiation markers. Expression levels of *Bglap, Col1a1, Runx2, and Alpl* were significantly decreased in day 10 *Vasn*^−/−^ osteoblast cultures (Fig. 5A-F). Expression levels of hypoxia and TGFβ-related genes were unaffected (Fig. 5I-K)**.**

### 3.5 The vitamin D endocrine system is severely disturbed

**Fig. 6:**
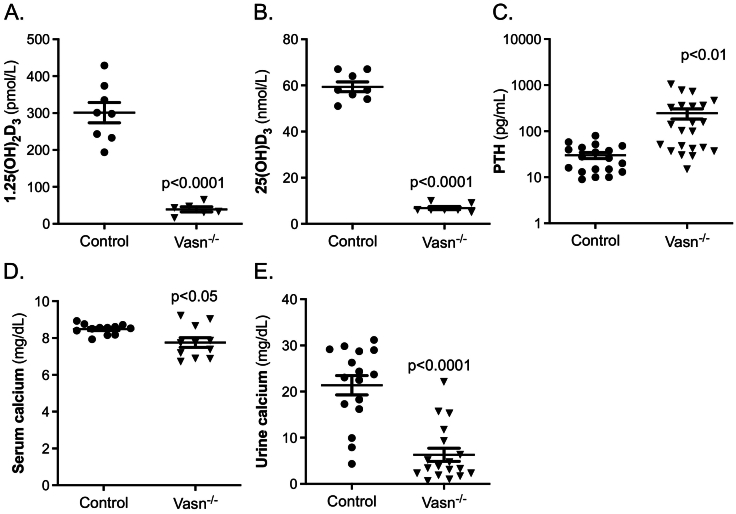
Calcium homeostasis markers determined in serum of control and *Vasn*^−/−^ mice. (A) 1,25(OH)_2_D_3_ (*n* = 6–8), (B) 25(OH)D_3_ (n = 6–8), (C) parathyroid hormone (PTH) (*n* = 23–26), (D) serum calcium, (E) urinary calcium (*n* = 14–17).

Next, we studied the effect of *Vasn* deficiency on the bone and calcium homeostasis regulatory hormones vitamin D and PTH. *Vasn*^−/−^ mice had extremely low levels of 1,25(OH)_2_D_3_ (Fig. 6A), which was also previously reported by Andrique *et al*, 2024 (Andrique et al., 2024). In addition, we demonstrated that Vasn^−/−^ mice are deficient in 25(OH)D, the precursor of 1,25(OH)_2_D_3_ (Fig. 6B).

Serum PTH measurement demonstrated strongly elevated PTH levels (8-fold increase) in *Vasn*^−/−^ mice (Fig. 6C). Serum calcium level was marginally and borderline significantly lower in *Vasn*^−/−^ mice (Fig. 6D) while urine calcium concentration was significantly diminished by about 65 % (Fig. 6E), suggesting reduced uptake of calcium in the intestine, which is supported by lower *Trpv6* expression in the intestine (Supplementary Table 2).

Since vitamin D levels were extremely low in the *Vasn*^−/−^ mice, we measured the expression of liver and renal enzymes involved in vitamin D synthesis and breakdown. The liver enzymes *Cyp3a11* and *Cyp27a1* were significantly reduced in *Vasn*^*−/−*^ mice, whereas vitamin D 25-hydroxylase (*Cyp2r1*) was not affected (Supplementary Table 2). In the kidney, the key enzyme involved in synthesis, 1α-hydroxylase (*Cyp27b1*) was unaffected, whereas 24-hydroxylase (*Cyp24*), involved in the degradation of 25(OH)D and 1,25(OH)_2_D_3_, was slightly upregulated by *Vasn* deficiency (Supplementary Table 2).

**Fig. 7:**
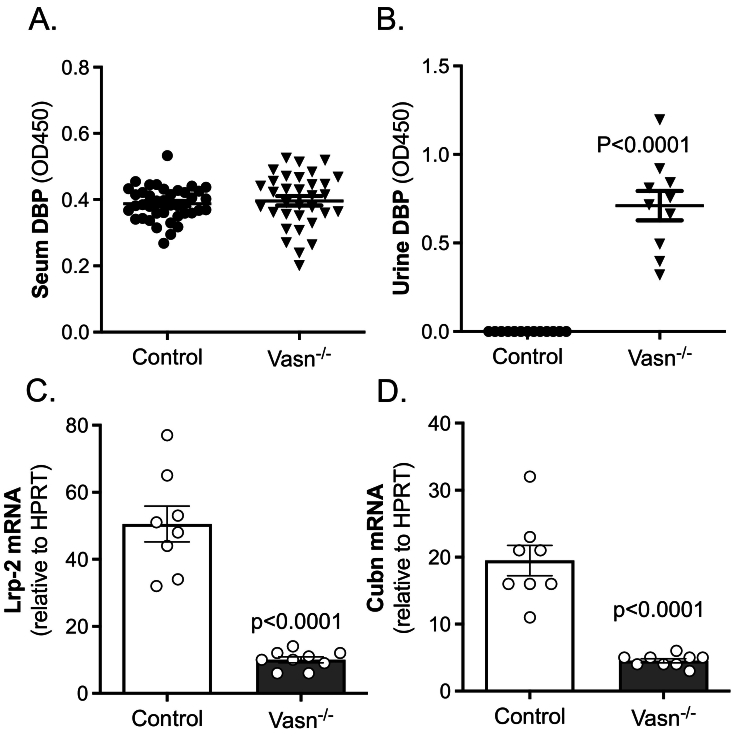
Vitamin D binding protein (DBP) measurements in serum and urine of control and *Vasn*^−/−^ mice. (A) serum DBP, (B) urinary DBP (*n* = 10–13). Renal mRNA expression of (C) megalin (*Lrp2*) and (D) cubulin (*Cubn*), corrected by normalization for the housekeeping gene *Hprt* (n = 8–9).

To further examine the vitamin D endocrine system in *Vasn*^−/−^ mice, we measured serum levels of vitamin D_3_ binding protein (DBP). This revealed unchanged serum DBP levels in *Vasn*^−/−^ mice (Fig. 7A). However, we detected significant amounts of urinary DBP in *Vasn*^−/−^ mice, whereas in control mice, no urinary DBP could be detected (Fig. 7B). Additional mRNA expression analysis in the kidney revealed strongly reduced levels of DBP carrier proteins megalin (*Lrp2*) and cubulin (*Cubn*) in *Vasn*^*−/−*^ mice (Fig. 7C and D). Moreover, *Vasn*^*−/−*^ mice suffered from severe proteinuria and histological assessment of renal tissue demonstrated malformed glomeruli (see Supplementary Fig. 2).

### 3.6 Phosphate homeostasis is unaffected despite disturbed expression of key-regulators

**Fig. 8:**
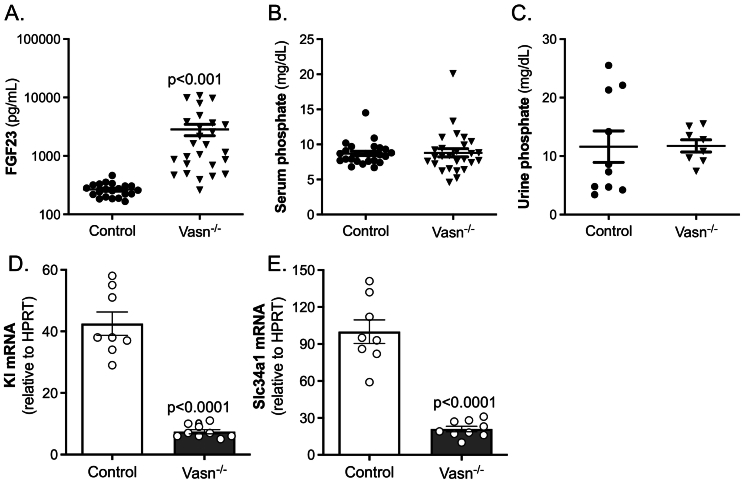
Phosphate homeostasis markers in serum and urine of control and *Vasn*^−/−^ mice. (A) serum Fgf23 (*n* = 26–29), (B) urinary phosphate (n = 8–18), (C) serum phosphate (*n* = 27–36). Renal mRNA expression of (C) klotho (*Kl*) and (D) sodium-dependent phosphate transport protein 2 A (*Slc34a1*), corrected by normalization for the housekeeping gene *Hprt* (n = 8–9).

FGF23 is a bone-derived hormone that enhances renal phosphate excretion and inhibits 1,25-(OH)_2_D_3_ production in the kidney. In *Vasn*^*−/−*^ mice, Fgf23 levels were strongly elevated (12-fold, Fig. 8A), while both urinary and serum phosphate levels were unaltered compared to control mice (Figs. 8B and C). Klotho (Kl) is a kidney-derived protein crucial for FGF23 action (Kurosu et al., 2006). In *Vasn*^−/−^ kidneys *Kl* mRNA levels were reduced to approximately 20 % of that of control mice (Fig. 8D). The expression of the sodium/phosphate co-transporter (*Slc34a1*) was strongly reduced as well (Fig. 8E).

## 4 Discussion

The current study demonstrates that vasorin is highly expressed in the skeleton during fetal development and is present in neonatal bone with high expression in periost, endost and osteocytes. Loss of vasorin leads to strongly reduced bone mass and trabecular connectivity. This is paralleled by reduced osteoblast differentiation and mineralization in *ex vivo* bone marrow cultures while *ex vivo* osteoclast formation and resorptive capacity was unaffected. These data demonstrate the involvement of Vasn in bone metabolism, which is in line with the recent proposal by Andrique *et al*, 2024 (Andrique et al., 2024).

### 4.1 Vasn skeletal localization and Vasn^−/−^ mice bone phenotype

Localization studies in *Vasn*^*lacZ*^ knock-in reporter mice has shown that Vasn is abundantly expressed in the skeleton, including osteoblasts, osteocytes and osteoclasts but absent in the bone marrow compartment. Vasn deficiency did not affect bone marrow-derived osteoclast formation and resorbing activity, while osteoclast activity was modestly reduced *in vivo*. This discrepancy can be best explained by the absence of a coupling or circulating factor in the cell cultures. The reduced osteoblast differentiation and mineralization *in vitro* mimicks the low levels of bone formation (P1NP) in serum of the *Vasn*^−/−^ mice, suggesting that an osteoblast defect appears to drive the low bone mass phenotype in *Vasn*^*−/−*^ mice, which corresponds to other murine models. In mice lacking Lamin A/C and in a murine model of accelerated senescence reduced bone formation is coupled to a lower bone turnover with secondary reduced osteoclast activity (Jilka et al., 1996; Li et al., 2011).

### 4.2 Vasn^−/−^ mice have elevated FGF23 but normal phosphate levels

Interesting is the expression of Vasn in osteocytes. These bone cells have come more and more into focus with respect to controlling bone metabolism as well as mineral homeostasis (Teti and Zallone, 2009; Dallas et al., 2013). An osteocyte-specific product, the phosphatonin Fgf23 (Shimada et al., 2004; Feng et al., 2006) is strongly increased in *Vasn*^*−/−*^ mice. This suggests that Vasn inhibits Fgf23 production/release in osteocytes. If the primary effect of *Vasn* deficiency on Fgf23 would originate in the kidney and be related to 1,25(OH)_2_D_3_, the low 1,25(OH)_2_D_3_ levels would have led to low and not to the observed high FGF23 levels.

The extremely high FGF23 levels in *Vasn*^*−/−*^ mice would kick off a strong change in phosphate levels. Due to its phosphaturic action one would have expected a strong increase in urinary and decrease in serum phosphate. However, these levels are not significantly different between wild type and Vasn-deficient animals. Apparently, the phosphate homeostatic system functions properly to maintain unchanged serum phosphate levels in the absence of Vasn. This may be explained by the strongly reduced renal klotho and phosphate transporter NaPi2A mRNA expression. Klotho is essential for the FGF23 action (Kurosu et al., 2006). Klotho acts as a co-receptor for FGF23 in the kidney to trigger FGF receptor signaling leading to reduction in phosphate reabsorption (Kurosu et al., 2006). The low renal klotho level in the *Vasn*^*−/−*^ mice therefore will likely prevent the high levels of FGF23 to be overactive. In addition, the reduced NaPi2a expression will limit excessive excretion of phosphate due to the high Fgf23 levels.

### 4.3 Mice lacking *Vasn* display diminished serum vitamin D

The *Vasn*^−/−^ mice have an extremely low 1,25(OH)_2_D_3_ level. The most well-known mouse model with an extremely low 1,25(OH)_2_D_3_ level is the 1α-hydroxylase-deficient (*Cyp27b1*^*−/−*^) mouse (Dardenne et al., 2001). They share several characteristics with mice lacking Vasn, including hypocalcemia and high PTH levels as well as reduced bone formation/turnover. However, the bone phenotype is much more severe compared to that in the *Vasn*^−/−^ mice. A striking difference between the *Vasn*^*−/−*^ and *Cyp27b1*^*−/−*^ mice is the Fgf23 level being very high and very low, respectively (Safadi et al., 1999). Although FGF23 is known to inhibit renal CYP27B1 expression and 1,25(OH)_2_D_3_ production (Shimada et al., 2004), the low klotho mRNA levels in *Vasn*^*−/−*^ mice may prevent Fgf23 from causing low 1,25(OH)_2_D_3_ levels as observed by unaltered Cyp27b1 expression. Hence, it is unlikely that the low serum 1,25(OH)_2_D_3_ levels found in *Vasn*^*−/−*^ mice is due to the increased Fgf23 level.

Besides 1,25(OH)_2_D_3_, we also measured very low levels of 25(OH)D_3_, which constitutes a rather unique phenotype and may explain the low serum 1,25(OH)_2_D_3_. Low levels of both megalin and cubulin in *Vasn*^*−/−*^ mice suggests impaired tubular reabsorption of DBP. In fact, the most likely explanation for low serum 25(OH)D and 1,25(OH)_2_D_3_ levels is the profound elevation of urinary DBP, which binds vitamin D metabolites and should be undetectable in the urine. Such a mechanism of losing 25(OH)D_3_ *via* the urine has been described for megalin deficiency (Nykjaer et al., 1999). Megalin deficiency in mice leads to the failure of tubular reabsorption of DBP-25(OH)D_3_ complexes, resulting in loss of DBP and 25(OH)D_3_ *via* the urine and low serum 1,25(OH)_2_D_3_ levels. Mice lacking DBP share several characteristics with the *Vasn*^−/−^ mice as well, including diminished serum levels of 25(OH)D_3_ and 1,25(OH)_2_D_3_ and reduced bone formation (Safadi et al., 1999). However, there is also an important difference between the models, namely having no (*Dbp*^−/−^ mice) and having normal (*Vasn*^−/−^ mice) serum DBP levels. Related to this, *Dbp*^−/−^ mice have no vitamin D-deficient phenotype (hypocalcemia and hypophosphatemia), due to normal levels of free circulating vitamin D, which is the bioactive fraction and usually reflects <1 % of the total vitamin D in the circulation (Safadi et al., 1999).

The feedback loop mechanism to safeguard calcium homeostasis seems to function properly in *Vasn*^−/−^ mice. The extremely low 1,25(OH)_2_D_3_ levels will consequently lead to reduced intestinal Ca uptake, which is reflected by the lower serum calcium level. This in turn leads to the observed increased PTH levels and an increased renal calcium reabsorption as reflected by the lower urinary calcium concentration. Nevertheless, these mice have a significantly lower serum calcium concentration.

Although a detailed renal phenotypic analysis was not performed, initial histological analyses demonstrated an impact of *Vasn* deficiency on renal structure, including glomerular capillary ectasia and collapsed glomerular capillary tufts (Supplementary Fig. 2). This may explain the observed low serum calcium, low 1,25(OH)_2_D_3_, and 25(OH)D_3_ levels and the impaired renal DBP handling and implicate a role of *Vasn* in proper renal development and function.

Mice lacking Vasn start to die 4 weeks after birth, which constitutes a limitation of the model, as it prevents the ability to study the function of this protein during postnatal development, sexual maturation and aging. The exact cause underlying their premature death requires further studies.

## 5 Conclusion

Vasn is a protein directly involved in bone metabolism. In addition, Vasn is implicated in vitamin D metabolism and calcium/phosphate homeostasis. The function of Vasn in bone seems to be confined to the osteoblast/osteocyte lineage and in the production of FGF23 and phosphate homeostasis. The precise molecular processes as well as the etiology of the renal phenotype and calcium and vitamin D metabolism in mice lacking Vasn requires further analysis.

## CRediT authorship contribution statement

**Marco Eijken:** Writing – review & editing, Writing – original draft, Visualization, Validation, Supervision, Project administration, Methodology, Investigation, Formal analysis, Data curation, Conceptualization. **A. Michaela Krautzberger:** Methodology, Investigation, Formal analysis, Data curation, Conceptualization. **Manuela Scholze-Wittler:** Methodology, Investigation, Formal analysis, Data curation, Conceptualization. **Bianca Boers-Sijmons:** Methodology, Investigation, Formal analysis, Data curation. **Marijke Koedam:** Project administration, Methodology, Investigation, Formal analysis, Data curation. **Barbara Kosiol:** Project administration, Methodology, Investigation, Formal analysis, Data curation. **Heinrich Schrewe:** Writing – review & editing, Supervision, Resources, Project administration. **Johannes P. van Leeuwen:** Writing – review & editing, Writing – original draft, Supervision, Resources. **Bram C. van der Eerden:** Writing – review & editing, Writing – original draft, Visualization, Methodology, Investigation, Formal analysis, Data curation.

## Declaration of competing interest

The authors declare that they have no known competing financial interests or personal relationships that could have appeared to influence the work reported in this paper.

## Data Availability

Data will be made available on request.

## Appendix A Supplementary data

Supplementary Fig. 1. Generation of *Vasn*^−/−^ and *Vasn*^*flox/flox*^ mice. (A) Targeting strategy showing the *Vasn* wild type allele and the targeting vector. The *Vasn*^*neo-flox*^ allele was generated by homologous recombination introducing a *loxP*-flanked selection cassette (neo) upstream and a third loxP site downstream of the coding region on exon 2. Subsequent Cre recombination either produced a *Vasn*^*flox*^ allele after excision of the selection cassette or a *Vasn*^*null*^ allele in which both the selection cassette and the coding region had been excised. The introduction of an additional *HindIII* site (H) allowed for discrimination of the different alleles via Southern blot analysis using external 5′ and 3′ probes. (B, C) Southern blot analysis of HindIII-digested genomic DNA from a *Vasn* wild type (*Vasn*^*+/+*^) clone, a targeted *Vasn*^neo-flox/+^ clone carrying one wild type and one *Vasn*^neo-flox^ allele, a *Vasn*^flox/+^ clone carrying one wild type and one *Vasn*^*flox*^ allele and a *Vasn*^null/+^ clone carrying one wild type and one *Vasn*^null^ allele using an external 5′ probe (B) and 3′ probe (C). Location of HindIII sites and size of DNA fragments after HindIII digestion are indicated in (A). (D) PCR genotyping results used for discrimination of *Vasn* wild type (+/+), heterozygous (*+/−)*, and homozygous knock-out (−/−) mice. Forward and reverse primers are indicated as black arrows in (A). (E) Northern blot analysis demonstrating the absence of *Vasn* RNA expression in a homozygous *Vasn*^*−/−*^ (−/−) compared to a heterozygous *Vasn*^*+/−*^ (*+/−*) adult mouse kidney (left). Ethidium bromide-stained gel showing rRNA bands as loading control (right). Supplementary Fig. 2. Kidney Phenotype of *Vasn*^−/−^ mice. (A, B) Representative images of hematoxylin and eosin-stained kidney sections from 4-week-old mice. (A) Comparison of histology of renal cortex between wild-type and knock-out mice. (B) Higher magnification reveals glomerular capillary ectasia and numerous dilated tubules filled with proteinaceous casts (asterisk and black arrows, respectively; left) as well as completely collapsed glomerular capillary tufts (right) in *Vasn*^−/−^ mice. (Magnification of objective: A: 10×, B: 100×). (C) Sodium dodecyl sulfate polyacrylamide gel electrophoresis (SDS-PAGE) analysis of urinary proteins in 2 μl urine from 25-day-old mice demonstrated massive proteinuria in moribund *Vasn* knock-out mice. Albumin standards (BSA) were loaded as control. Image 1

Supplementary tables Image 2
