# Supplementary material for: Vasorin-deficient mice display disturbed vitamin D and mineral homeostasis in combination with a low bone mass phenotype
Source: Bone Rep. 2024 Jul 18;22:101792. doi: 10.1016/j.bonr.2024.101792 (PMC11326953; doi:10.1016/j.bonr.2024.101792)
Supplement: Supplementary file 1 — Supplementary Fig. 1. Generation of Vasn−/− and Vasnflox/flox mice. (A) Targeting strategy showing the Vasn wild type allele and the targeting vector. The Vasnneo-flox allele was generated by homologous recombination introducing a loxP-flanked selection cassette (neo) upstream and a third loxP site downstream of the coding region on exon 2. Subsequent Cre recombination either produced a Vasnflox allele after excision of the selection cassette or a Vasnnull allele in which both the selection cassette and the coding region had been excised. The introduction of an additional HindIII site (H) allowed for discrimination of the different alleles via Southern blot analysis using external 5′ and 3′ probes. (B, C) Southern blot analysis of HindIII-digested genomic DNA from a Vasn wild type (Vasn+/+) clone, a targeted Vasnneo-flox/+ clone carrying one wild type and one Vasnneo-flox allele, a Vasnflox/+ clone carrying one wild type and one Vasnflox allele and a Vasnnull/+ clone carrying one wild type and one Vasnnull allele using an external 5′ probe (B) and 3′ probe (C). Location of HindIII sites and size of DNA fragments after HindIII digestion are indicated in (A). (D) PCR genotyping results used for discrimination of Vasn wild type (+/+), heterozygous (+/−), and homozygous knock-out (−/−) mice. Forward and reverse primers are indicated as black arrows in (A). (E) Northern blot analysis demonstrating the absence of Vasn RNA expression in a homozygous Vasn−/− (−/−) compared to a heterozygous Vasn+/− (+/−) adult mouse kidney (left). Ethidium bromide-stained gel showing rRNA bands as loading control (right). Supplementary Fig. 2. Kidney Phenotype of Vasn−/− mice. (A, B) Representative images of hematoxylin and eosin-stained kidney sections from 4-week-old mice. (A) Comparison of histology of renal cortex between wild-type and knock-out mice. (B) Higher magnification reveals glomerular capillary ectasia and numerous dilated tubules filled with proteinaceous casts (asterisk and [file mmc1.pptx]

## Slide 1
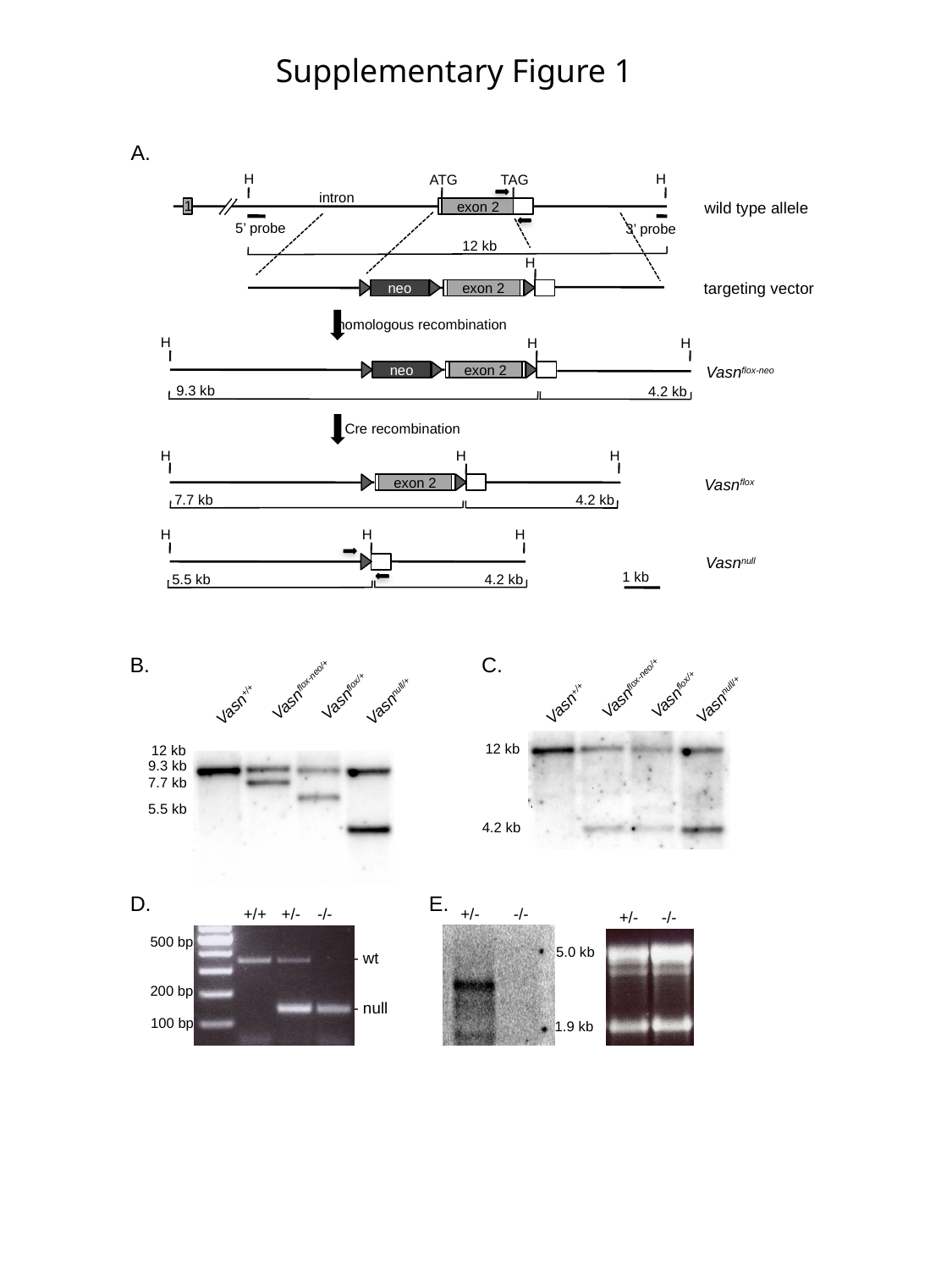

Supplementary Figure 1
A.
H
H
ATG
TAG
intron
1
exon 2
5’ probe
3’ probe
12 kb
H
neo
exon 2
wild type allele
targeting vector
homologous recombination
H
H
H
Vasnflox-neo
neo
exon 2
9.3 kb
4.2 kb
Cre recombination
H
H
H
Vasnflox
exon 2
7.7 kb
4.2 kb
H
H
H
Vasnnull
1 kb
5.5 kb
4.2 kb
B.
C.
Vasnflox-neo/+
Vasnflox-neo/+
Vasnflox/+
Vasnflox/+
Vasnnull/+
Vasnnull/+
Vasn+/+
Vasn+/+
12 kb
12 kb
9.3 kb
7.7 kb
5.5 kb
4.2 kb
D.
E.
+/+
+/-
-/-
500 bp
- wt
200 bp
- null
100 bp
+/-
-/-
+/-
-/-
5.0 kb
1.9 kb

## Slide 2
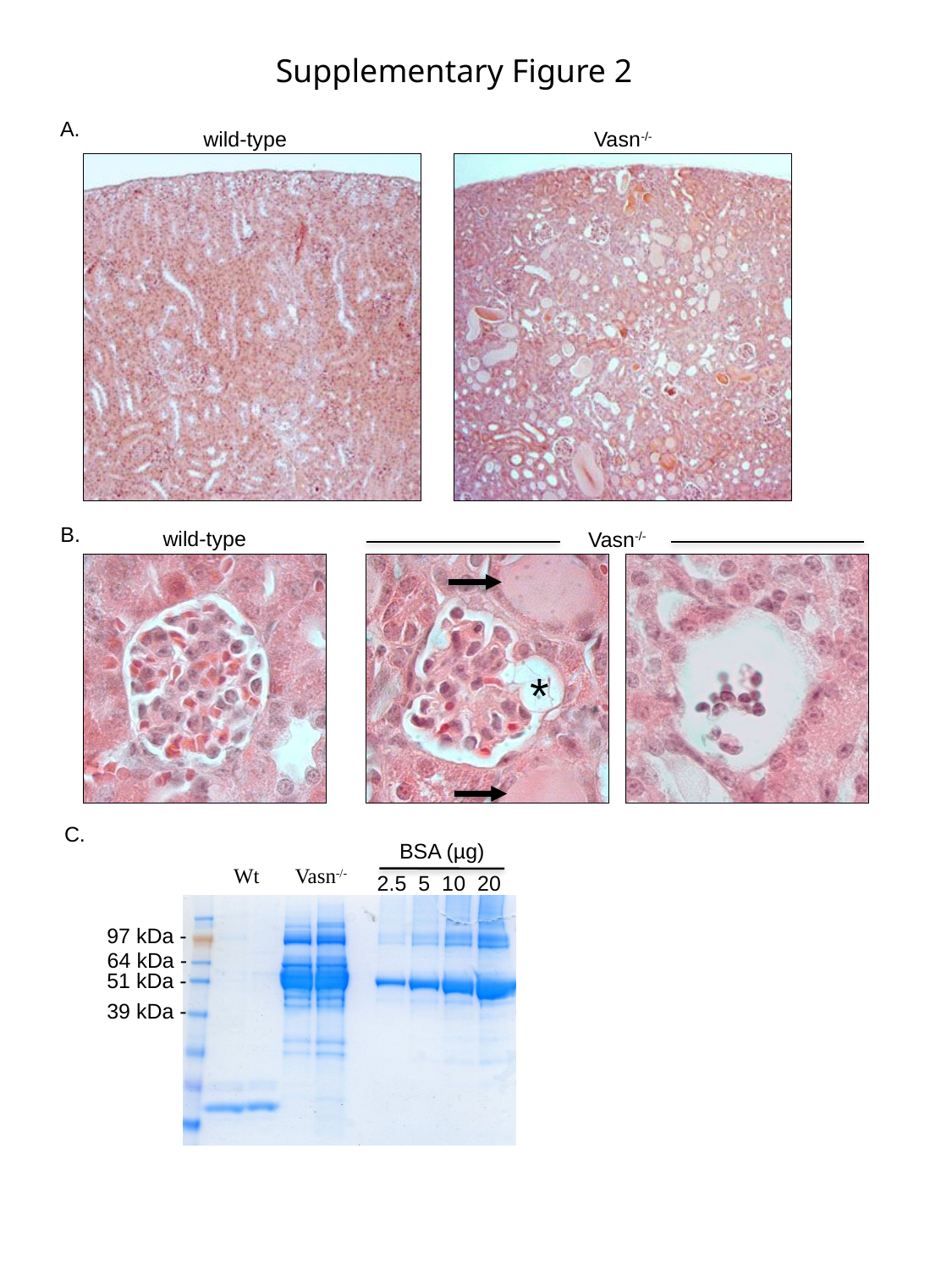

Supplementary Figure 2
A.
wild-type
Vasn-/-
B.
wild-type
Vasn-/-
*
C.
BSA (µg)
Wt
Vasn-/-
2.5 5 10 20
97 kDa -
64 kDa -
51 kDa -
39 kDa -
