# Supplementary material for: Vasorin-deficient mice display disturbed vitamin D and mineral homeostasis in combination with a low bone mass phenotype
Source: Bone Rep. 2024 Jul 18;22:101792. doi: 10.1016/j.bonr.2024.101792 (PMC11326953; doi:10.1016/j.bonr.2024.101792)
Supplement: Supplementary file 2 — Supplementary tables [file mmc2.docx]

**Supplementary information belonging to the manuscript entitled:**

Vasorin-deficient mice display disturbed vitamin D and mineral homeostasis in combination with a low bone mass phenotype

by Eijken M et al.,

**Supplementary Table 1.** Primer sequences used for qPCR analysis

| **Gene** | **Forward primer 5’-3’** | **Reverse primer 5’-3’** |
| --- | --- | --- |
| *Alpl* | ACACTCGGCCGATCGGGACT | CGCCACCCATGATCACGTCGA |
| *Bglap* | CCTGAGTCTGACAAAGCCTTCAT | CAAGGTAGCGCCGGAGTCT |
| *Col1a1* | ACGGCTGCACGAGTCACA | CCAAGGGAGCCACATCGAT |
| *Cubn* | GCAAAGAGTTTAGCCTGCGG | AATTGGCAGGGTAGTTGGGG |
| *Cyp24a1* | GGCTCTTTGCTGGATAATCCAA | GCTAGGTACCAGGATGCCAAGAT |
| *Cyp27a1* | AGGAGGGCAAGTACCCAATAAGA | TTGCTCTCCTTGTGCGATGAAG |
| *Cyp27b1* | CCCAGCTGCCCCTGTTAAA | GTTTCCTACACGGATGTCTCTGTCT |
| *Cyp2r1* | CACTTTGATGAAAAGTACTGGAAAGATC | CCAAGACAATGTCTTCTCCCTAGAG |
| *Cyp3a11* | CAGAGAAGTAAATTGCTGACAAACAAG | GTTTACGAGTCCCATATCGGTAGAG |
| *Hif1a* | ACACACAGAAATGGCCCAGTGAGA | TCGTCCTCCCCCGGCTTGTT |
| *Hprt* | tctcgatttcctatcagtaacagcat | aaatacagccaacactgctgaaac |
| *Kl* | AAAGAGTCCACGCCAGACATG | CTCGGGCTTAAGAACAGACTCAGT |
| *Klf10* | GGTGTGGCAAGACTTACTTTAAAAGTT | AGCGAGCAAACCTCCTTTCA |
| *Lrp2* | TTGGCCCAATGGACTCACTC | GCGCTCTATTTTCTGCAGGC |
| *Runx2* | aagtgcggtgcaaactttct | tctcggtggctggtagtga |
| *Slc34a1* | CAACAGAGGCTTCCACTTCTATGTC | CATTTCTCCATGGTGGTGTTTG |
| *Smad7* | GCGGAAACCGGGGGAACGAA | AGCCTCCCCACGCGAGTCTT |
| *Sod1* | CGATGAAAGCGGTGTGCGTGC | TGGTTCACCGCTTGCCTTCTGC |
| *Trpv6* | TTCCAGCAACAAGATGGCCTCTACTCTGA | ATCCGCCGCTATGCACA |
| *Vasn* | CCAGAGGTGAAGGACTGAGGC | CAGGAGAGGTGGCAGGCAGC  S100 |
| *S100g* | CCTGCAGAAATGAAGAGCATTTT | CTCCATCGCCATTCTTATCCA |

**Supplementary Table 2.** Gene expression levels of enzymes involved in vitamin D biosynthesis and breakdown in the liver (n=11), kidney (n=9) or intestine (n=9) of control and *Vasn^-/-^* mice. Gene expression was corrected by normalization to the housekeeping gene *Hprt.*

|  |  | Control | Vasn^-/-^ | p-value |
| --- | --- | --- | --- | --- |
| *Kidney* |  |  |  |  |
| *Cyp24a1* |  | 0.64±0.15 | 1.22±0.16 | <0.05 |
| *Cyp27b1* |  | 0.034±0.005 | 0.084±0.023 | 0.070 |
| *Trpv5* |  | 4.6±0.75 | 2.4±0.47 | <0.05 |
| *Liver* |  |  |  |  |
| *Cyp2r1* |  | 0.67±0.08 | 0.54±0.07 | 0.052 |
| *Cyp3a11* |  | 263±31 | 127±25 | <0.01 |
| *Cyp27a1* |  | 42±4 | 23±2 | <0.001 |
| *Intestine* |  |  |  |  |
| *Trpv6* |  | 2315±391 | 554±188 | <0.001 |
| *S100g* |  | 16±3.6 | 2.7±0.35 | <0.01 |
